# Bone and Energy Metabolism Parameters in Professional Cyclists during the Giro d’Italia 3-Weeks Stage Race

**DOI:** 10.1371/journal.pone.0042077

**Published:** 2012-07-27

**Authors:** Giovanni Lombardi, Patrizia Lanteri, Rosa Graziani, Alessandra Colombini, Giuseppe Banfi, Roberto Corsetti

**Affiliations:** 1 I.R.C.C.S. Istituto Ortopedico Galeazzi, Milano, Italia; 2 CEDAL Lab, Gallarate, Italia; 3 Chair of Clinical Biochemistry, School of Medicine, University of Milano, Milano, Italia; 4 Liquigas Cannondale pro-cycling team, Medical board, Sesto al Reghena, Italia; Universidad Europea de Madrid, Spain

## Abstract

Cycling is a not weight-bearing activity and is known to induce bone resorption. Stage races are really strenuous endurance performances affecting the energy homeostasis. The recently highlighted link, in the co-regulation of bone and energy metabolism, demonstrates a central role for the equilibrium between carboxylated and undercarboxylated forms of osteocalcin. Aim of this study was to understand the acute physiological responses to a cycling stage race in terms of bone turnover and energy metabolism and the possible co-regulative mechanisms underlying their relationship. We studied nine professional cyclists engaged in 2011 *Giro d’Italia* stage race. Pre-analytical and analytical phases tightly followed academic and anti-doping authority’s recommendations. Bone and energy metabolism markers (bone alkaline phosphatase, tartrate-resistant acid phosphatase 5b, total and undercarboxylated osteocalcin, leptin and adiponectin) and related hormones (cortisol and testosterone) were measured, by Sandwich Enzyme Immunoassays, at days -1 (pre-race), 12 and 22 during the race. The power output and the energy expenditure (mean and accumulated) were derived and correlated with the biochemical indexes. During the race, bone metabolism showed that an unbalance in behalf of resorption, which is enhanced, occurred along with a relative increase in the concentration of the undercarboxylated form of osteocalcin that was indirectly related to the enhanced energy expenditure, through adipokines modifications, with leptin decrease (high energy consumption) and adiponectin increase (optimization of energy expenditure). The exertion due to heavy effort induced a decrease of cortisol, while testosterone levels resulted unchanged. In conclusion, during a 3-weeks stage race, bone metabolism is pushed towards resorption. A possible relationship between the bone and the energy metabolisms is suggested by the relative correlations among absolute and relative concentrations trends of undercarboxylated OC, adipokines concentrations, BMI, fat mass (%), power output and the derived energy expenditure.

## Introduction

Professional cycling 3-weeks stage races such as *Tour de France*, *Vuelta a España* and *Giro d’Italia*, very popular sport events, could be included among the most strenuous athletic performances [1]. These races last 21–22 days with more than 3000 km covered and only one or two days of rest; athletes are submitted to very intense metabolic effort, combining aerobic and anaerobic metabolism, particularly during mountain stages and time trials [2], [3], [4]. Thus, studies concerning the metabolic changes occurring during these competitions are of particular interest for describing the biochemical pathways orchestrating the energy expenditure required to perform the mechanical work, as highlighted by previous studies on endurance athletes [5], [6].

Although, papers concerning hormonal, haematological and biochemical changes during cycling races are available [7], [8], the link between bone and energy in cycling has never been investigated.

The modification of the hormonal profile during the *Vuelta a España* was described in detail [2], [7], [9]. Morning serum concentrations of testosterone, FSH, LH and cortisol, in nine cyclists during the *Vuelta* 1999, measured before the race (at the end of the first, the second and the third week of competition) showed no variations in gonadotropins, and a decrease in cortisol and testosterone as a result of long duration and intense exercise [2]. Interestingly, thyroid hormones, which are known to be involved in adaptation of an organism to physical exercise [9], were modified during a *Vuelta.* Hormones were measured in 16 cyclists before the race and at the end of each of the three weeks of competition: free 3,5,3′-triiodothyronine, total and free-thyroxine significantly increased during the last part of the race, while TSH and total 3,5,3′-triiodothyronine remained unchanged. The increase in thyroid hormones might be due to haemoconcentration or to the release of thyroid hormone-transporting proteins. These effects could be also related to an impaired peripheral conversion of thyroxine to 3,5,3′-triiodothyronine [9], possibly brought by cortisol increase that is in turn induced by leptin decrease [10], as a result of an energy unbalance. This response is typical of the hardest part of the race, when mountain stages, requiring high energy expenditure, are common [2], [11]. The intensity and the duration of the exercise during 3-weeks stage races induce a rise of insulin growth factor I concentrations during the first week, and, after an adaptation, its stabilization after three weeks. This parameter is linked to nutritional status and to both insulin and growth hormone production and release [7].

Bone metabolism, especially bone formation, is known to be enhanced by exercise. Physical activity is indeed recommended for assuring correct bone density and homeostasis, and to prevent bone mineral depauperation. Bone mineral density (BMD), assessed in athletes practicing different sports, showed general positive effect, but with wide differences between disciplines, especially when considering distinct bone districts, genders and weight-bearing exercise level. On the other hand, no univocal data have been obtained from bone metabolism markers, although it is evident that, at least long-lasting loads increase the circulating levels of formation markers [12], [13].

Cycling is characterized by a low level of skeletal load, with the training specifically based on the not-weight-bearing bike riding performed across the whole year. As a consequence, cyclists have lower BMD than runners [14] and age-matched controls [15]; furthermore, bone health status in adolescent cyclists is affected by the heavy training and the exercise in absence of load [16].

Bone markers were never studied in long-term cycling performances. Therefore, no data about the possible bone metabolism changes during a high-demanding 3-weeks stage race exist. Noteworthy, a modern approach to bone function is claiming its active role in regulating energy metabolism [17]. The regulation of osteoblasts and osteoclasts activity has recently been integrated into an endocrine perspective, which includes a consistent link with energy metabolism and sympathetic nervous system. The endocrine function of the bone is exerted through the release of a series of hormones and, among them, osteocalcin (OC), a molecule with a role in local bone physiology, but also able to regulate fat and glucose metabolism, insulin secretion and pancreatic β cells proliferation; moreover, in adipocyte, OC induces the release of adiponectin which, in turn, reduces insulin resistance [12], [17], [18]. OC is a 5.8 kDa, hydroxyapatite-binding, protein exclusively synthesized by osteoblasts, odontoblasts and hypertrophic chondrocytes. It possesses 3 vitamin-K dependent, γ-carboxyglutamic acid residues (Gla-OC), which are responsible for the calcium binding properties of the protein. There are some post-transcriptional forms of OC. In energy metabolism pathway, it is of particular interest the undercarboxylated form (Glu-OC) which could be directly released, by osteoblasts, in balance with the carboxylated form [17], [18], or released from the main OC molecule, when the bone environment is acidified by acid phosphatase activity, expression of activation of osteoclasts [19]. Circulating Glu-OC acts on insulin-sensitive tissues and pancreatic β cells [18].

The control of OC secretion is assured by sympathetic tone, which is in turn controlled by leptin, an adipocyte-derived hormone, which can regulate osteoblasts: its action is mediated through two different neural pathways. Leptin concentrations are not influenced by short exercise, but are decreased by long-term exercise or by exercise with high energy expenditure; adiponectin, instead, presents a delayed increase. In general, long-term exercise could decrease leptin and increase adiponectin. Adiponectin concentrations are negatively related with body fat, fasting plasma insulin and oral glucose tolerance and positively related with glucose disposal. No data concerning leptin and adiponectin concentrations at rest and after exercise in cyclists exist [20]. Leptin and adiponectin correlate positively and negatively with fat mass, respectively in normal and overweight subjects. Among professional athletes, cyclists appear to have the lowest body mass index (BMI), fat percentage and adipokines concentrations, especially during long-term training and competition. BMI in cyclists could significantly change during a 3-weeks stage race [11]: this finding should be taken into account for interpreting metabolism modifications.

The regulation of energy metabolism in cyclists during a high-demanding and long-lasting race is crucial for maintaining a sufficient level of performance for three weeks. This study is aimed to study bone metabolism markers, adipokines and hormones involved in the loop of energy metabolism regulation in professional cyclists who competed in the 2011 *Giro d’Italia*. The choice of cyclists involved in a 3-week stage race, in order to study the integrative physiology of bone and energy metabolism, was derived from the evidence of peculiar body composition and accelerated bone turnover of these athletes associated with the extreme energy demands necessary to carry out the race.

## Materials and Methods

### Ethics Statement

The study was approved by the Reference Ethical Committee (ASL Milano 1). The athletes involved in this study have given written consent for sample collection and data analysis. Clinical investigations were conducted according to the principles expressed in the Declaration of Helsinki.

### Subjects

Nine professional cyclists belonging to the Liquigas-Cannondale team were recruited. They were involved in the *Giro d’Italia 2011* and followed across the race, from May 8^th^ to May 29^th^.

Athletes covered 3524.5 km and the mean speed was 35.6 km/h. The route characteristics are reported in Table 1.

**Table 1:** Characteristics of the route.

Day	Stage	Stage Lenght (km)	Level Difference (m)	Kind of Stage	Blood drawing
−3	/	/	/	/	Official Antidoping
−2	/	/	/	/	
−1	/	/	/	/	Study
1	1	19.3	96	Time Trial	
2	2	244.0	780	Flat	
3	3	173.0	2340	Flat	
4	4	216.0	2779	Medium Mountain	
5	5	191.0	3637	Medium Mountain	Official Antidoping
6	6	216.0	3200	Flat	
7	7	110.0	2796	Medium Mountain	
8	8	217.0	912	Flat	
9	9	169.0	5412	Mountain	
10	Rest	/	/	/	
11	10	159.0	882	Flat	
12	11	144.0	3097	Medium Mountain	Study
13	12	184.0	463	Flat	
14	13	167.0	4664	Mountain	
15	14	210.0	6643	Mountain	
16	15	229.0	9490	Mountain	
17	Rest	/	/	/	
18	16	12.7	756	Time Trial	
19	17	230.0	5336	Medium Mountain	
20	18	151.0	1949	Flat	Official Antidoping
21	19	209.0	3569	Mountain	
22	20	242.0	3464	Mountain	Study
23	21	31.5	67	Time Trial	

In the Table are reported the correspondence between day, length (km), level difference (m) of each stage along with the kind of stage and the blood sampling performed for this study and the blood sampling for official anti-doping testing.

Mean athletes’ age was 26.7±2.5 years. Weight and height were measured in the morning before the stage in fasting conditions and body mass index (BMI) was calculated as weight/(height)^2^; anthropometrical parameters are reported in Table 2.

No drugs or supplements influencing the iron metabolism were taken by athletes; only non-steroidal anti-inflammatory drugs and antibiotics were administered where needed. The *Giro* 2011 was a “no-needle” race: therapies and drugs were allowed only for evident diseases. Diet was strictly controlled by team physicians and was composed by a 45% of carbohydrates, 36% of proteins and 19% of lipids. The caloric intake was balanced on the base of the energy expenditure; it was set at 6000 kcal/day and kept for the whole study.

Blood drawings were performed on the day before the start (day -1), and on days 12 and 22 of the race (Table 1).

At day -1 athletes performed 3 h of light training at 55% VO_2_max including a short bout (30 min) of medium-high levels sub-threshold commitment at 60% VO_2_max. The diet on day -1 had the same composition of that carried out during the race.

### Power Output and Net Energy Expenditure Measurements

The individual power output was measured for each stage, through the power sensor PowerMeter™ (SRM GmbH, Jülich, Germany) integrated within the bike pedal (sensitivity ±2%), as previously described [21]. Briefly, the PowerMeter™ utilizes 8 strain gauges on 16 grids, within a Wheatstone’s bridge: when the force is applied on the pedals the resistance to the transmission impresses a pressure on the measuring bridge. The pressure intensity proportionally modifies the strain gauge length that is translated in a change of the electrical resistance and thus in a proportional variation of the output frequency. The instantaneous torque (τ), calculated as the output frequency subtracted of the basal frequency, and the instantaneous angular velocity (ω) are related to the power output (P) as follow:


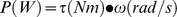


The PowerMeter™ automatically derived the instantaneous energy expenditure (kcal) from the instantaneous power output by applying the following unit conversion equivalence:


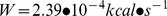


The total energy expenditure is given by the sum of the instantaneous expenditures.

**Table 2:** Anthropometric and power-related measurements across the stage race.

Parameters	day -1	day 12	day 22	P-value
Height (m)	1.82±0.05	/	/	/
Weight (kg)	69.1±5.24^a^	68.9±4.94^a^	67.4±5.06^b^	<0.05
Mean power output (W)	/	214.3±14.96^a^	256.2±14.63^b^	<0.001
Mean power output/Weight (W/kg)	/	3.1±0.17	3.7±0.16	<0.001
Accumulated power output (W)	/	1929.0±134,60^a^	2305,5±131,69^b^	<0.001
Accumulated power output/Weight (W/kg)	/	27.9±1.56	33.4±1.43	<0.001
Mean net energy expenditure (kcal)	2213.1±156.01^a^	3401.5±249.41^b^	3755.9±239.96^c^	<0.001
Accumulated net energy expenditure (kcal)	2213.1±156.01^a^	30613.4±2244.66^b^	33803.0±2159.62^c^	<0.001

In the Table are reported the results of the measurements at the same time-points of blood sampling, with the exception of the height that is reported only for day -1.

Mean power output and accumulated power output, as both raw data and corrected for body weight, refer to the mean or the sum, respectively, of the consecutive measurements relative to the period preceding the blood sampling. The same applies to mean net energy expenditure and accumulated net energy expenditure for whom the rest values (day -1) correspond to the basal metabolic rate. Measurements are expressed as mean±SD and values bearing different letters in apex are significantly different (p<0.05).

### Blood Drawings

Pre-analytical warnings were strictly followed [22] to avoid any possible factor affecting the analytical phase [23]. Particularly, International Cyclist Union (UCI) and World Anti-Doping Agency (WADA) guidelines for collection and transport of specimens were followed [24].

Blood was drawn between 08^00^ and 10^00 ^h in fasting subjects resting in bed ten minutes after their awaking. Evacuated tubes (BD Vacutainer Systems, Becton–Dickinson, Franklin Lakes, NJ, USA) were used for analytes measurement (BD K_2_EDTA 3.5 mL and 7.0 mL plain tubes, BD SSTII *Advance*). Immediately after drawing, tubes were inverted ten times and stored in a sealed box at 4°C. Controlled temperature was assured during transportation: a specific tag (Libero Ti1, Elpro, Buchs, Switzerland) was used for temperature measurement and recording. Samples were transported by car or by both train and car: the time elapsed from drawing to laboratory was 1 h 30 min at day -1, 7 h 50 min at day 12, and 1 h 30 min at day 22. The differences in delay were due to the sampling performed in places at different distances from the laboratory. Such delays do not affect the analytical output for the measured parameters [25].

The K_2_EDTA-anticoagulated blood was homogenized for 15 min prior to be analyzed, as recommended by UCI and WADA [24].

Official anti-doping controls were performed, by antidoping agencies, prior the start of the race (day -3) and during the final phases (day 20); moreover, one of the recruited subjects was submitted to an additional check at day 5 (Table 2).

### Renal Function

Renal function was monitored by using creatinine and cystatin C. Estimated glomerular filtration rate (eGFR) was calculated by different equations based on these two parameters [26].

### Analytes Measurement

Total Osteocalcin (Gla-OC) and undercarboxylated Osteocalcin (Glu-OC) were measured, in plasma, by Sandwich Enzyme Immunoassays (EIA - Takara Bio Inc., Otsu, Shiga, Japan) using specific monoclonal antibodies directed against the different forms of OC, following the manufacturer specifications. The specificity was 0.5 ng/mL for the Gla-OC assay kit and 0.25 ng/mL for the Glu-OC assay kit, while the within-run and between-run coefficients of variation (CV) were 4.8% and 2.4% for Gla-OC and 6.7% and 9.9% for Glu-OC.

The percentage ratio of Glu-OC relative to the total OC was calculated as:


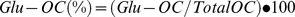


Bone-specific alkaline phosphatase (BAP) and Tartrate-resistant Acid Phosphatase 5b (TRAP5b) activities were measured in serum by immunocapture methods (Quidel Corporation, San Diego, CA, USA) based on the conversion of the uncolored p-nitrophenylphosphate in its yellowish-colored dephosphorylated form, the p-nitrophenol in alkaline or acidic environment, respectively. The sensitivity of BAP assay kit was 0.7 U/L and CVs were 5.8% and 7.6%. The sensitivity of TRAP5b was 0.2 U/L and CVs were 2.2% and 3.0%.

Adiponectin and leptin concentrations were measured in serum by ELISA using specific capture monoclonal antibodies (R&D Systems Inc., Minneapolis, MN, USA). Briefly, according to manufacturer specifications, microplates were coated for 18 hours with the capture antibodies (2.0 µg/ml for adiponectin and 4.0 µg/ml for leptin), washed 3 times with 0.05% Tween 20 in phosphate buffered saline, pH 7.3, and blocked for 1 hour with 1% bovine serum albumin in phosphate buffer, pH 7.3. Following 3 washes, diluted samples (1∶10) in 1% bovine serum albumin in phosphate buffer, pH 7.3, were added in duplicates and incubated for 2 hours. The horseradish peroxidase-conjugated polyclonal detection antibodies (2.0 µg/ml for adiponectin and 12.5 ng/mL for leptin) (R&D Systems Inc., Minneapolis, MN, USA) were added and incubated for 2 hours. Following 3 washes, a solution of 1∶1 H_2_O_2_-Tetramethylbenzidine was added for 20 min and then the reaction was stopped with 1N H_2_SO_4_. All steps were performed at RT. The absorbance were read at λ = 450 nm (VICTOR™ X^3^, Perkin Elmer, Waltham, MA, USA). The analytes concentrations in samples were calculated against their respective standard curves. All samples and standards were evaluated in duplicate.

Serum cortisol and testosterone concentrations were tested on a Bayer Elecsys 2010 (Bayer AG, Leverkusen, Germany). Sensitivities were 0.018 µg/dL for cortisol and 0.025 ng/mL for testosterone, while CVs were 2.8% and 3.4%, respectively.

During the study the analyzers were regularly calibrated and controlled by both internal and external quality control schemes; moreover, all analytes were assayed in a single batch and by the same technician to limit analytical variability.

### Statistical Analysis

Statistical analysis was performed by GraphPad Prism v5.0 software (GraphPad Software Inc., LaJolla, CA, USA). All values in the descriptive analysis are expressed as the mean ± SD. Normal distribution of values were assayed by Kolmogorov-Smirnov normality test, while one-way Analysis of Variance (ANOVA) for repeated measures, with the Bonferroni’s correction, was used to compare data over time. Paired comparisons were performed by two-tailed t test. In the case of not normally distributed values, repeated measures were compared with the Kruskal-Wallis test with the Dunns’ correction. Correlation analysis was performed by the two-tailed Pearson correlation test (Spearman’s test for not normally distributed values); the same test was conducted to evaluate the correlation between the trends of these parameters across the time-points. The significance level was set at 0.05.

## Results

### Anthropometric and Power Measurements

Body weight significantly decreased in the second phase of the race, with the 22^nd^ day significantly differed from day -1 and day 12 (Table 2). Consequently, the BMI at day 22 (20.4±1.06) was significantly lower than those calculated for day -1 (20.9±1.27, p<0.001) and day 12 (20.8±1.14, p<0.001).

To characterize the metabolic effort in which the athletes are involved, the power output and the net energetic expenditure were derived. Values are reported in Table 2. The mean of all the measurements relative to the first half of the race was significantly lower than those of the second phase (p<0.001) as well as the sum of all the measurement (accumulated power output) until day 11 compared with those accumulated until day 21 (p<0.001). Even for what concern the net caloric expenditure, an increase was evident throughout the race with significant differences among the basal value, the mean and the accumulated expenditures until the 12^th^ and the 22^nd^ day.

Both the mean and the accumulated power outputs strongly correlated with the accumulated and the mean net energy expenditures (r = 0.95, p<0.001), and when they are corrected for body weight, this relationship is approximate to perfect fit (r = 0.99, p<0.001).

### Renal Function

No significant differences were reported on the serum parameters and also in glomerular filtration rate estimated using the Chronic Kidney Disease-Epidemiology Collaboration (CKD-EPI) creatinine equation, as previously reported [26].

### Bone Metabolism Markers

**Figure 1:**
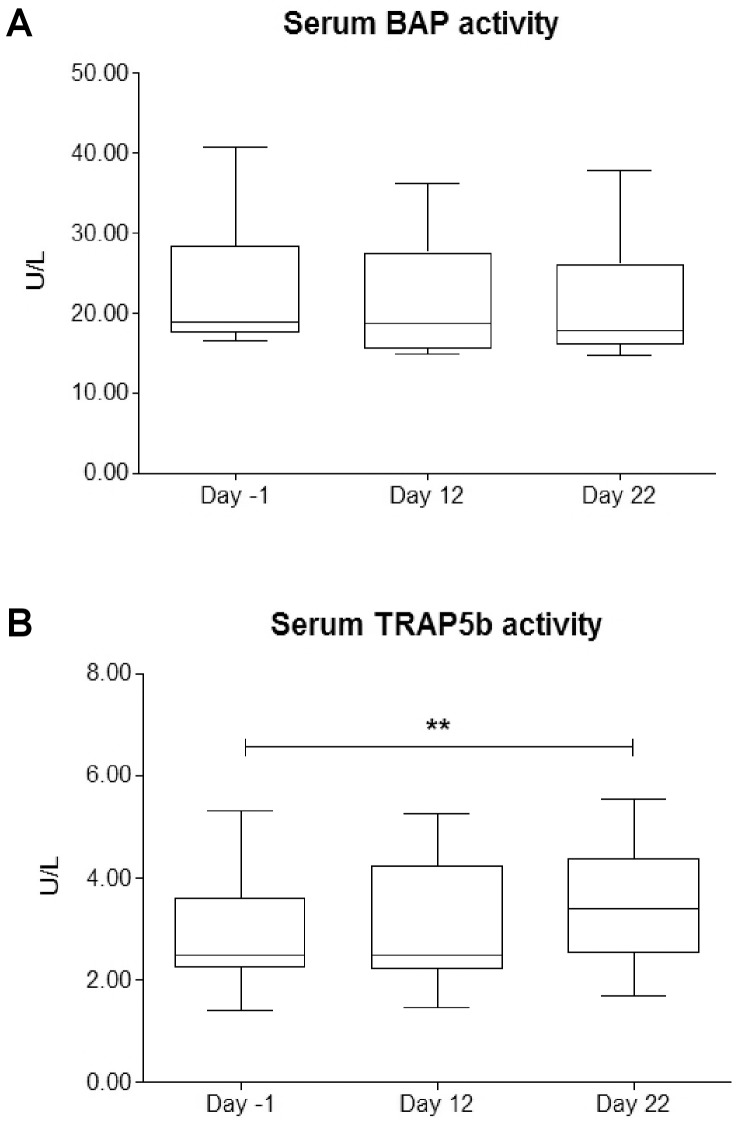
Modification of serum bone markers activity over the race. The Figure shows the trends of serum BAP (panel A) and serum TRAP5b (panel B) over the stage race. ** indicates a significant variation (p<0.01).

The trend of the bone metabolic markers is illustrated in Figure 1. BAP was unchanged (Figure 1A), whilst TRAP5b increased in the final part of the race respect to day -1 (p<0.01) (Figure 1B).

To give a numerical evaluation of the difference in metabolic activity of osteoblast and osteoclast, the ratio between the main metabolic indexes of these cells (BAP/TRAP5b) was calculated. The BAP/TRAP5b ratio significantly decreased from 8.3±2.51 at day -1 to 7.4±2.33 at day 12 (p<0.05) and to 6.5±2.10 at day 22 (p<0.001).

A fair correlation was found between BAP and TRAP5b trends (r = 0.44, p<0.05), while no correlation was found with the indexes of energetic expenditure and power output.

### Osteocalcin

**Figure 2:**
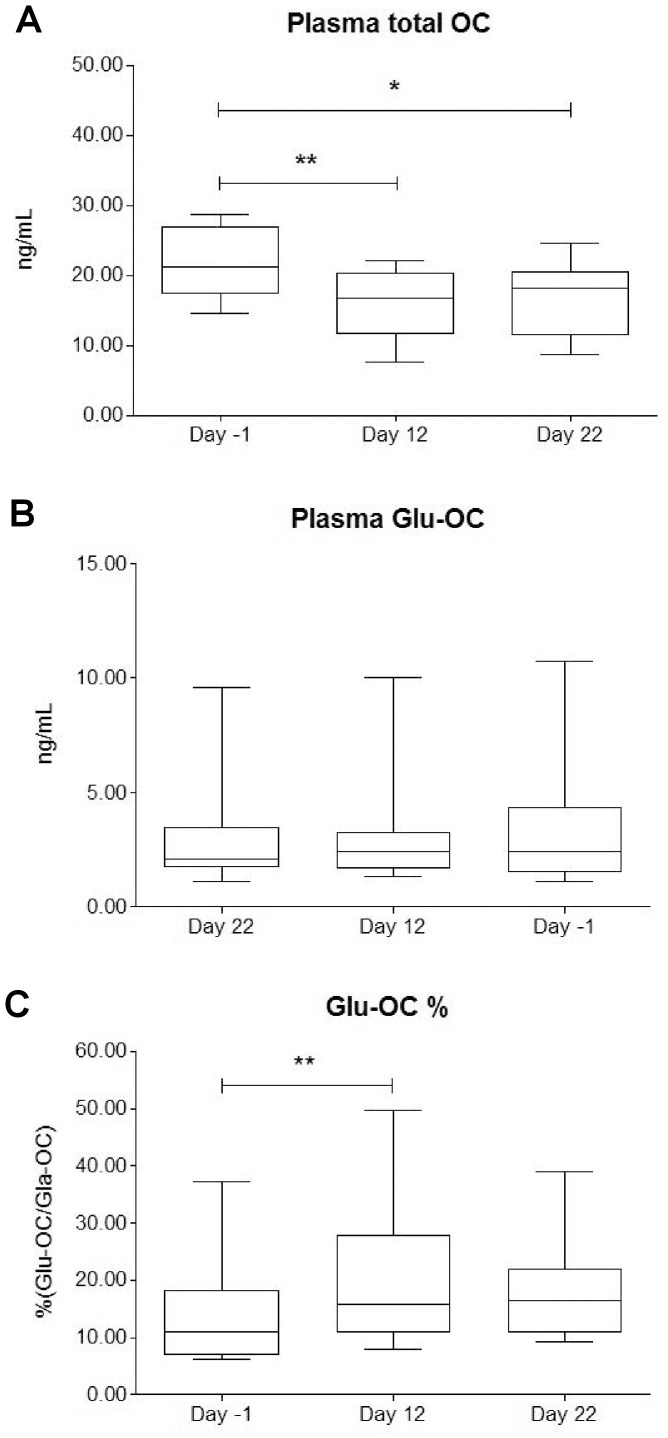
Modification of plasma osteocalcin levels and relative concentration of Glu-OC over the race. The Figure shows the trends of plasma total OC (panel A), plasma Glu-OC (panel B) and relative percentage concentration of Glu-OC (panel C) over the stage race. ** and * indicate significant variations (p<0.01 and p<0.05, respectively).

The data of total OC and Glu-OC are illustrated in Figure 2. Total OC decreased along the race (p<0.01 between day -1 and day 12, p<0.05 between day -1 and day 22) (Figure 2A). Absolute Glu-OC concentrations, at the contrary, were unchanged during the race, but they showed a higher inter-individual variability (Figure 2B).

The relative concentration of Glu-OC, i.e. the percentage of total OC represented by Glu-OC, increased in the first part of race (p<0.01) (Figure 2C).

Moderate to good correlations were found between TRAP5b and total OC (r = 0.50, p<0.05), total OC and Glu-OC (r = 0.51, p<0.05), Glu-OC and Glu-OC percentage (r = 0.80, p<0.001); total OC fairly correlated with BAP/TRAP5b ratio (r = 0.37, p<0.05). Total OC correlated with mean and accumulated power outputs in a fair indirect manner (r = −0.44, p<0.05 for raw data, and r = −0.46, p<0.05 for weight corrected power outputs) and inversely with accumulated and mean net energy expenditures (r = −0.44, p<0.05).

### Energy Metabolism Markers

**Figure 3:**
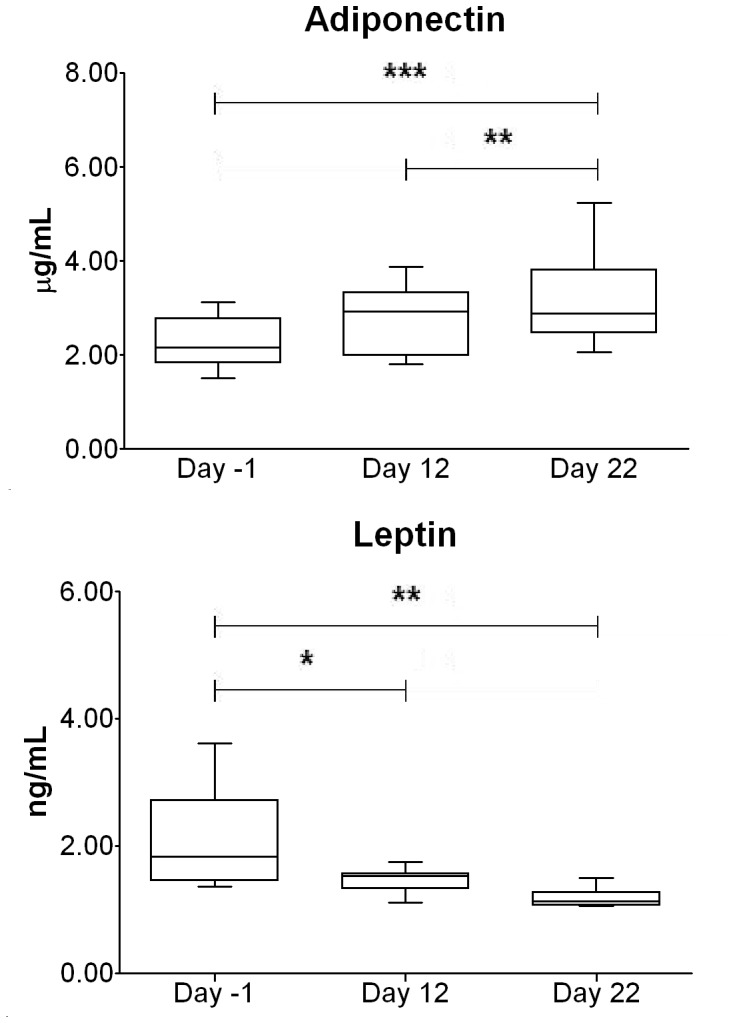
Modification of adipokines levels over the race. The Figure shows the trends of serum adiponectin (panel A) and serum leptin (panel B) over the stage race. ***, ** and * indicate significant variations (p<0.001, p<0.01 and p<0.05, respectively).

Adipokines behavior during the race is shown in Figure 3. While adiponectin constantly rose (p<0.001 between day -1 and day 22, p<0.01 between day 12 and day 22) (Figure 3A), leptin steadily decreased (p<0.05 between day -1 and day 12; p<0.01 between day -1 and day 22) (Figure 3B).

Adiponectin and leptin were inversely fairly related (r = −0.41, p<0.05) and they fairly correlated with the BAP/TRAP5b index (r = 0.30, p<0.05; r = 0.32, p<0.05, respectively), while only adiponectin correlated with total OC in a fair indirect manner (r = 0.37, p<0.05). Adiponectin, but not leptin, and fairly correlated with Glu-OC (r = 0.30, p<0.05). Of note, the GluOC (%) directly fairly correlated with BMI (r = 0.41, p<0.05) and the fat mass percentage (r = 0.48, p<0.05), while absolute concentrations of GluOC fairly correlated with fat mass percentage (r = 0.45, p<0.05). Parallel, adiponectin, but not leptin, showed a direct moderate correlation with BMI (r = 0.51, p<0.001).

For what concern the mean and the accumulated power outputs they both equally correlated with the energy markers adiponectin (r = 0.44, p<0.05) and, inversely, but more strongly, with leptin (r = −0.72, p<0.001). These correlations were kept when the power outputs were adjusted on body mass.

The energy expenditures indexes showed a good correlation, directly, with adiponectin (r = 0.51, p<0.01) and inversely with leptin (r = −0.69, p<0.001).

### Hormones

**Figure 4:**
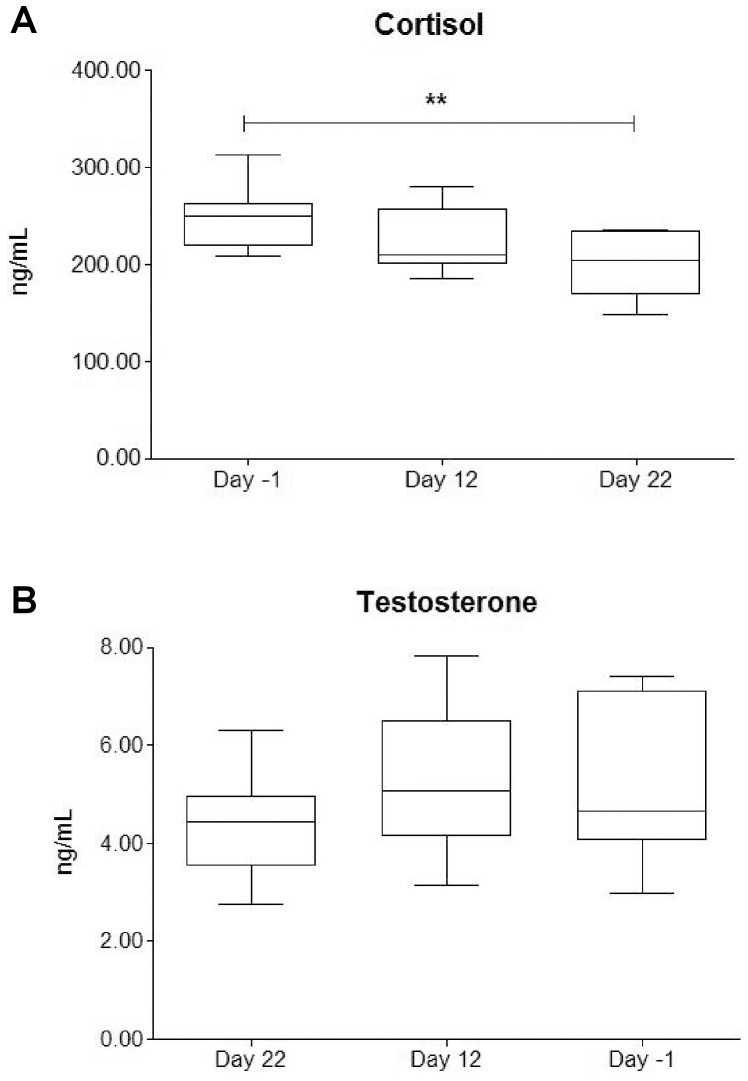
Modification of cortisol and testosterone concentrations over the race. The Figure shows the trends of serum cortisol (panel A) and serum testosterone (panel B) over the stage race. ** indicates a significant variation (p<0.01).

Testosterone concentration was unchanged during the race (Figure 4A), whereas cortisol concentration at day 22 were decreased in respect to day -1 (p<0.01) (Figure 4B).

No differences in the ratio cortisol/testosterone were observed throughout the study; the values attested at 51.5±16.57 at day -1, 44.6±14.86 at day 12 and 48.3±15.25 at day 22.

Cortisol was directly fairly related with GlaOC (r = 0.41, p<0.05) and moderately with leptin (r = 0.55, p<0.01) and body mass (r = 0.56, p<0.01). Testosterone was directly moderately correlated with body weight (r = 0.56, p<0.01). Moreover, cortisol, but neither testosterone nor cortisol/testosterone, was inversely related with accumulated and mean power outputs, both as raw data (r = −0.45, p<0.05) and corrected for weight (r = −0.47, p<0.05). Finally accumulated and mean net energy expenditures were fairly inversely related to cortisol levels (r = −0.44, p<0.05).

## Discussion

Three-weeks cycling stage races represent unique and paradigmatic situations of long and strenuous daily competition [27], [28] and they represent ideal models for investigating the homeostatic responses to strenuous and continuative workload. Cycling is one of hardest sport discipline, owing to high demanding training and competition, long-lasting season, high level of competition and high selection within athletes that results in only about 800 professionals in the world. The 3-weeks stage races represent the highest level of fatigue [28], possibly affecting the athletes’ health.

A recent knowledge in endocrinology suggests a fundamental role of bone in energy metabolism. Bone is not a passive tissue but it is able to secrete hormones, and, among them OC, which act on pancreatic cells to regulate insulin and, consequently, glucose disposal and utilization in peripheral tissues, particularly in skeletal and cardiac muscles [17], [29]. Cycling is known to affect bone metabolism [14], even in young [16]: previous reports suggest that cycling could be dangerous for bone homeostasis and, consequently, it could affect all the bony-regulated functions. An unbalance between bone formation and resorption does not represent only a local issue but it could have systemic impacts.

Although the effects on bone metabolism are exerted during long periods of time, the strong effort of a 3-weeks stage race could be sufficient to induce valuable modifications. The stimulation of osteoblastic and/or osteoclastic function is dependent on exercise, but the response appears to be not immediate in experimental studies based on training periods. However, in professional athletes this delay should not be considered: the skeleton is continuously loaded/unloaded and stimulated [12]. At this purpose, it is important to underline that the *Giro* occurs in the month of May, after three months of training and two of training and competition.

In this study we tried to define a correlation among the indexes of bone metabolism, those of energy utilization and the measurement of effort within the context of one of the hardest cycling races.

The metabolic effort was characterized through the measurement of the power output and they were comparable with those previously reported for a six-stage road stage and the Tour de France [30], [31]. Only Vogt, in 2008 [32], recorded a 29% higher power output, but the data were referred to only the ascents of the Tour. To better correlate the indexes of metabolic effort with the physiological response, the power output (raw and corrected for body weight) and the net energy expenditure were calculated as the mean and the accumulated values relative to the period preceding the blood sampling (Table 2).

For what concern the bone metabolism, BAP activity remained stable (Figure 1A), testifying that osteoblast metabolism, and thus bone formation, are preserved during such a strenuous exercise. BAP activity decreased the day after an ultramarathon of 245 km and then recovered pre-race values after 3 and 5 days [33]. Throughout the training year BAP activity was reported to decrease in professional triathletes [34] and to increase in alpine skiers [13]. Thus, BAP activity is modified in long-term periods in athletes: a stage race could be too short for evidencing modifications or even, cyclists could have specific stability of bone formation parameters, as deducted from the narrow inter-individual variability in these athletes; however, data on this specific topic are lacking.

The bone resorption appear to be stimulated during the race as witnessed by the increase in TRAP5b activity more evident in the second phase of the race when, however, a higher inter-individual variability is present (Figure 1B). The resorption seems to be strictly connected to this specific continuous and stressful exercise, since it is not reported, after long periods of training, in top-level athletes of other disciplines [12], [34], [35] However, when TRAP5b is evaluated, its increase is evidenced, in top-level athletes, during the most intense part of the season [13]. The balance between the metabolic activities, operating within the bone remodeling unit, could be represented by the ratio between BAP and TRAP5b. During the race this ratio decreases, better demonstrating the imbalance towards the resorption. This trend, due to osteoclasts activation specifically marked by TRAP5b, which could enhance OC decarboxylation, may represent a common trait of high-demanding period in sportsmen, possibly inducing bone density lowering in cyclists [14], [36], [37].

Bone resorption and osteoclasts activity are undoubtedly enhanced in heavy cycling: the real impact of this finding and (or) possible prompt recovery should be, however, further investigated.

Adipokines have a direct and important role on bone and cartilage metabolism [38], [39]. The co-dependence of bone and energy metabolism has never been studied in professional athletes. Very few papers, describing chronic effects of exercise on adipokines, are available and none of such studies were conducted during strenuous long-lasting performances [20]. It is reported that while strong acute exercise lowers leptin concentration, adiponectin levels increased only after heavy long-lasting exercise through the reduction of body mass and fat [20]. The study of adipokines in professional cyclists is particularly interesting, because these athletes are characterized by low BMI and very low fat percentage [40].

Adiponectin increased throughout the race (Figure 3A), while leptin dramatically decreased, showing a progressive narrowing in standard deviation (Figure 3B). The two adipokines were inversely related, definitely demonstrating the rising need of energy during the race. This finding is also supported by the increase in the relative Glu-OC concentration (Figure 2C), which could act by stimulating the glucose utilization. Hinton and colleagues assessed the increase of OC in 5 elite male cyclists during a 6-days bicycle tour. They showed that OC increased of 300%, but this could be due to the very low OC levels at baseline and/or to the evaluation through only 5 days. Heavy increase in OC is likely due to the stressful initial mechanical stimulation exerted on the bone; finally, no information about the form of OC recognized by the assay were reported [41]. The energy metabolism is potently enhanced during the 3-weeks stage race: despite of the very high-caloric diet, the tremendous need of energy, testified by the increase in the net energy expenditure and the decrease in weight and, thus, in BMI (Table 2), stimulates adipokines and related bone metabolites. Moreover, adipokines trends were clearly related to the power output and the energy expenditures.

Even if the only correlation among adipokines and bone OC forms was found between adiponectin and Glu-OC, evident correlations were found among Glu-OC, Glu-OC (%) and fat mass (%) and BMI as well as between the adipokines and these anthropometrical features. These findings suggest the presence of a relation between undercarboxylated OC and the body composition in line with previous reports. However, we speculate the existence of another factor involved in their molecular link and further studies must be addressed to uncover this marker. This link is stressed by the relationship, even if fair, between the bony metabolic index BAP/TRAP5b and levels of adiponectin and leptin.

The decrease of cortisol (Figure 4A) was similar to that one previously described during *Vuelta* [11]. Cortisol trend should be linked to an exhausting mechanism, as suggested by the relationship the indexes of metabolic effort. At the same time, a decrease in endurance efforts and in adrenal sensitivity to adrenocorticotropic hormone stimulation, and a decreased hypothalamic-pituitary axis sensitivity to cortisol negative feedback, may occur [42]. Catabolic status, which is expected during a stage race, is usually marked by an increase of cortisol. In our study, despite the cortisol decrease, the catabolic status is, however, manifest and it is demonstrated, at the adipose tissue level, by the leptin decrease and, at the bony level, by the decrease in total OC (Figure 2) and BAP/TRAP5b. Remarkably, positive relationships were found among cortisol and leptin and among cortisol and total OC. A possible explanation of this phenomenon could be hide in the activation of the sympathetic nervous system, which is known to affect the physiological homeostasis.

We did not observe significant decrease in testosterone concentrations (Figure 4B), differently from a previous report [11] where testosterone constantly decreased through the three weeks of race of *Vuelta*, in18 cyclists belonging to two different professional teams. Testosterone was significantly different between the two teams, but the decreasing trend was observed for both [11]. This decrease was explained by body mass loss accompanied by the reduction in fat mass; similarly, in our study, we observed correlation between testosterone values and weight loss, despite the decrease of testosterone levels was not significant. Of note, our basal levels were lower than those reported before *Vuelta*, while similar during the competition. A previous study showed that testosterone values in professional cyclists, similar to ours, were no different from those in controls, who had a higher bone density [37]. We did not observe any correlation between cortisol, testosterone, cortisol/testosterone and bone markers, suggesting that the control of bone metabolism in cyclists is uncoupled from these hormones.

The variations of testosterone and cortisol in cyclists, even testifying a catabolic state, did not automatically lead to a decrease in performance or to a state of overtraining, as demonstrated in other sport disciplines [43]. Thus, further specific studies in cycling, during stage races and also during a whole season, should be necessary to better define hormonal modifications.

We finally want to point out that, to our knowledge, this is the first study in which the pre-analytical management of the samples has been accurately checked and, thus, we highlight a warning about the previous researches concerning the pre-analytical phase, mostly undescribed.

In conclusion, during a 3-weeks stage race, bone metabolism parameters showed an unbalance towards resorption. A possible relationship between bone and energy metabolisms is suggested by the relative correlations among absolute and relative concentrations trends of undercarboxylated OC, adipokines concentrations, BMI, fat mass (%), power output and the derived energy expenditure. The presence of this association, although a direct link cannot be demonstrated, supports the evidence of a strict involvement of bone in the regulation of the energy metabolism.
